# Immunoregulatory Intestinal Microbiota and COVID-19 in Patients with Type Two Diabetes: A Double-Edged Sword

**DOI:** 10.3390/v14030477

**Published:** 2022-02-25

**Authors:** Pavlo Petakh, Iryna Kamyshna, Andriy Nykyforuk, Rouan Yao, John F. Imbery, Valentyn Oksenych, Mykhaylo Korda, Aleksandr Kamyshnyi

**Affiliations:** 1 Department of Biochemistry and Pharmacology, Uzhhorod National University, 88000 Uzhhorod, Ukraine; pavlo.petakh@uzhnu.edu.ua (P.P.); djsunray@gmail.com (A.N.); 2 Department of Microbiology, Virology, and Immunology, I. Horbachevsky Ternopil National Medical University, 46001 Ternopil, Ukraine; 3 Department of Medical Rehabilitation, I. Horbachevsky Ternopil National Medical University, Majdan Voli 1, 46001 Ternopil, Ukraine; kamyshna_ii@tdmu.edu.ua; 4 Center of Molecular Inflammation Research, Department of Clinical and Molecular Medicine, Norwegian University of Science and Technology, 7491 Trondheim, Norway; rouan.yao@ntnu.no; 5 Institute of Clinical Medicine, University of Oslo, 0318 Oslo, Norway; j.f.imbery@medisin.uio.no; 6 Department of Medical Biochemistry, I. Horbachevsky Ternopil National Medical University, 46001 Ternopil, Ukraine; kordamm@yahoo.com

**Keywords:** type 2 diabetes, metformin, COVID-19, SARS-CoV-2, intestinal microbiota, immunoregulation

## Abstract

Coronavirus disease 2019, or COVID-19, is a major challenge facing scientists worldwide. Alongside the lungs, the system of organs comprising the GI tract is commonly targeted by COVID-19. The dysbiotic modulations in the intestine influence the disease severity, potentially due to the ability of the intestinal microbiota to modulate T lymphocyte functions, i.e., to suppress or activate T cell subpopulations. The interplay between the lungs and intestinal microbiota is named the gut–lung axis. One of the most usual comorbidities in COVID-19 patients is type 2 diabetes, which induces changes in intestinal microbiota, resulting in a pro-inflammatory immune response, and consequently, a more severe course of COVID-19. However, changes in the microbiota in this comorbid pathology remain unclear. Metformin is used as a medication to treat type 2 diabetes. The use of the type 2 diabetes drug metformin is a promising treatment for this comorbidity because, in addition to its hypoglycemic action, it can increase amount of intestinal bacteria that induce regulatory T cell response. This dual activity of metformin can reduce lung damage and improve the course of the COVID-19 disease.

## 1. Introduction

The human gastrointestinal (GI) tract is represented by an extremely diverse and complex microbial ecosystem with more than 10^14^ resident species interacting with the host and actively participating in many physiological processes. Such physiological processes include energy homeostasis, regulation of metabolism, synthesis of vitamins or other important molecules, immune responses, and finally metabolism of xenobiotics, toxins, carcinogens, and other harmful compounds [1,2,3,4,5]. It was found that changes in the intestinal microbiota are connected with various noncommunicable diseases—for example, type 2 diabetes (T2D), cardiovascular disease, GI diseases, and obesity [6,7,8,9,10].

The GI tract is an important target organ for the SARS-CoV-2 virus and possibly affects the severity of COVID-19 disease. GI symptoms, such as nausea, vomiting, and diarrhea, are common in COVID-19 patients. In addition, it was found that the SARS-CoV-2 virus penetrates the cell by binding its S-protein to the human angiotensin-converting enzyme 2 (ACE2) receptor, which, in particular, is widely expressed by intestinal enterocytes [11,12,13].

Evidence suggests diabetes mellitus is one of the most common comorbidities in COVID-19 patients, and pre-existing diabetes mellitus is considered as a risk factor for severe COVID-19 [14]. However, the underlying connection between the two is not fully established. Proinflammatory status, impaired innate immune response, increased ACE2 expression, vascular dysfunction, and prothrombotic status in patients with diabetes are potential contributors of increased vulnerability to SARS-CoV-2 infection and worsening prognostic outcome [15]. The composition of the microbiota in type 2 diabetes (T2D) patients varies, affecting the clinical course of COVID-19 [16]. In this review, we summarize various studies exploring mechanisms of T-lymphocyte modulation by the intestinal microbiota, the role of the gut–lung axis in COVID-19, and changes in the microbiota in T2D and COVID-19.

## 2. Immunoregulation, Gut Microbiota, and COVID-19

### 2.1. The Role of Intestinal Microbiota in T-lymphocyte Modulation

The first lines of defense against SARS-CoV-2 lie within the mucosa-associated lymphoid tissue (MALT)—namely, the nasal-associated lymphoid tissue (NALT) and the gut-associated lymphoid tissue (GALT), which are involved in inducing immune responses towards microorganisms. This is accomplished by promoting the differentiation and activation of immune cells, such as T helper 1 (Th1), Th2, dendritic cells (DCs), and macrophages [17]. Most of the CD4+ T cells are effector or memory T cells located in the intestinal lamina propria [18]. Upon activation by microbiota, antigens are presented by antigen-presenting cells (APCs), such as DCs, causing CD4+ T cells to differentiate into regulatory T cells (Tregs), Th1 cells, Th2, T_FH_, and Th17 cells [19,20].

Th1 cells are characterized by expression of the transcription factor T-bet, signal transducer and activator of transcription (STAT) 4, and the production of IL-2, IFN-γ, and tumor necrosis factor (TNF) β. They are involved in the cellular immunity and rejection process. In contrast, Th2 cells, which are mediators of humoral immunity, develop into IL-4-, IL-5-, and IL-13-producing cells by the transcription factor GATA-3 and STAT6. A new lineage of Th cells that selectively produce IL-17 has been proposed. This population, termed Th17, plays a critical role in the induction of inflammation and the pathogenesis of autoimmune diseases and rejection. Th17 cells are characterized by unique signaling pathways triggered by receptor-related orphan receptor (ROR) C2 or ROR-α. Th17 cells are involved in the host defense against bacteria, fungi, and viruses [21,22]. Th1, Th2, and Th17 cells are regulated by CD4 + CD25+ Tregs, which are important for the maintenance of peripheral tolerance [23,24]. Th17 cells and Tregs are extensively studied subsets of T cells, constituting a large proportion of the effector cells. The imbalance between the cells of these two subsets results in various disorders, ranging from inflammatory and autoimmune diseases to cancer [25].

Active viral replication was found in enterocytes of the small intestine [12]. Although the pathomechanism of GI lesions in SARS-CoV-2 infection was not fully studied, the authors did observe some integral loss of the intestinal barrier and dysbiosis in the microbiota. Violations of the integrity of the intestinal barrier activate the nonspecific and adaptive immune responses, which release pro-inflammatory cytokines into the circulatory system [26].

Dysbiosis can be defined as a reduction in microbial diversity and a combination of (1) the loss of beneficial bacteria, such as Bacteroides strains and butyrate-producing bacteria such as Firmicutes and (2) a rise in pathobionts (symbiotic bacteria that become pathogenic under certain conditions), including Gram-negative *Escherichia coli* of the phylum Proteobacteria [27]. Furthermore, dysbiosis can prompt multiple immune disorders mediated by T cells [28]. The T cell functions are dysregulated in many patients with severe COVID-19 [29,30]. Qin et al. showed that severely affected patients have fewer Tregs (specifically, induced Tregs) [31]. Several studies have also shown that GI tract dysbiosis can alter the Treg/CD4+ T cell axis, and this may result in a pathogenic outcome [32]. Secondly, T cells are exhausted in severe COVID-19 patients versus non-severe COVID-19 patients [29,30]. Thus, T cell exhaustion in severely affected patients may be a combinatorial effect of hyper-inflammation and imbalanced GI microbiota.

Among the vast number of commensal bacteria inhabiting the GI tract, some species of key immunoregulatory bacteria play an important role in controlling the maturation of various T-lymphocyte subpopulations. T-cell receptors (TCRs) of these cells, in turn, are commensal-specific [33]. One of the main functions of the intestinal microbiota is to modulate T-lymphocytes in the GALT. This modulation is carried out in two primary ways, either by individual microorganisms or a consortium of microorganisms working together or by special microbial metabolites. In the following sections, we focus on these two mechanisms in more detail.

#### 2.1.1. Induction of T-Regulatory Cells (Treg) by Microorganisms and Their Consortia

**Figure 1:**
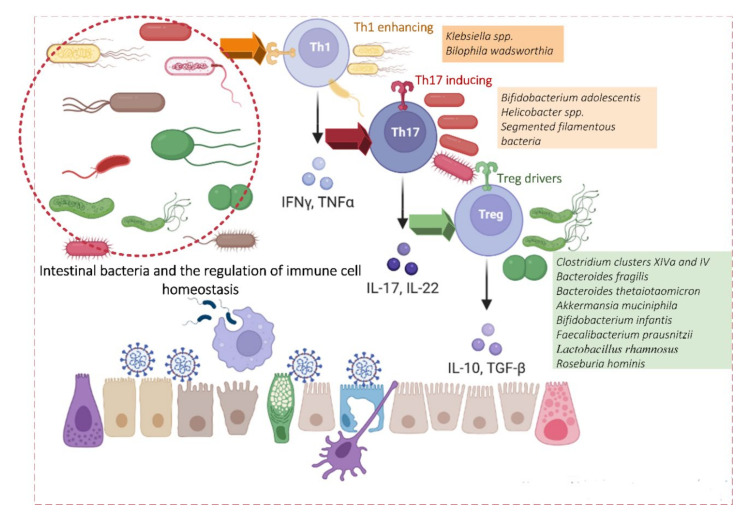
Intestinal bacteria and immune cell homeostasis regulation. The main bacterial inducers of intestinal CD4+ T cells are shown.

Treg cells are important for maintaining immune homeostasis by suppressing an excessive immune response. Two main groups of Tregs have been identified: natural Tregs (nTregs) and peripherally induced Tregs (pTregs). Several consortia of *Clostridia* species have been identified as potent intestinal Treg inducers [34,35] (Figure 1). For example, colonization by altered Schaedler flora (ASF), which includes *Lactobacillus acidophilus*, *Lactobacillus murinus*, *Bacteroides distasonis*, *Mucispirillum schaedleri*, *Eubacterium plexicaudatum*, a fusiform-shaped bacterium (ASF 356), and two Clostridium species (ASF 356, ASF 502) all induce expansion of Tregs [36].

Clostridial cluster IV and XIVa co-induce transforming growth factor-beta (TGFβ) secretion by epithelial cells in the intestine, resulting in pTreg differentiation and proliferation in the large intestine [35,37]. Recently, intestinal induction of pTreg after colonization by an inherently more complex mouse microbiota, Oligo-MM12, has also been described [38]. Oligo-Mouse-Microbiota (Oligo-MM12), a community of 12 strains representing members of the major bacterial phyla in the murine gut, is another microbial composition that is proving useful in evaluating how the microbiota influences disease development and therapeutics [39].

Among the 19 clusters (I to XIX) of the class *Clostridia* living in the intestine, the representatives of clusters IV and XIVa, known as *Clostridium leptum* and *Clostridium coccoides,* respectively, have the greatest potential of inducing Tregs [35]. *Clostridium* cluster XIVa includes species belonging to the *Clostridium*, *Ruminococcus*, *Eubacterium*, *Coprococcus*, *Roseburia, Lachnospira, Dorea*, and *Butyrivibrio* genera. Clostridium cluster IV is composed of the *Clostridium*, *Ruminococcus, Anaerofilum*, and *Eubacterium* genera [40]. Treg-inducing activity was detected in mice following colonization by a 46-strain cocktail of the genus *Clostridium* [35]. Additionally, a cocktail of 17 human strains of *Clostridia* that induced Treg activity has also been identified and described [34].

Species *Faecalibacterium prausnitzii* (one of the representatives of *Clostridium* cluster IV) affects the balance of Th17/Treg in the GALT [41]. Th17/Treg balance is important for maintaining immune homeostasis, as Th17s cause autoimmunity and inflammation, while conversely, Tregs suppress these pathological processes [42]. *F. prausnitzii* is one of the key butyrate producers in the intestine and can decrease inflammation by stimulating the production of IL-10 and blocking activation of nuclear factor NF-κB [43]. This leads to inhibition of pro-inflammatory cytokines (IFN-γ, TNF-α, IL -1β, IL-8, IL-12) and increased activity of Foxp3+ Tregs in the GALT [43]. In 2016, a protein called microbial anti-inflammatory molecule (MAM) was identified in *F. prausnitzii* culture supernatants [44]. At least seven peptides of the protein MAM induced in vitro production of IL-10 and blocked the development of DSS-induced colitis in mice in vivo [45].

Representatives of the phylum *Bacteroidetes*, *Bacteroides fragilis* and *Bacteroides thetaiotaomicron*, are also inducers of Treg differentiation in the gut [46]. Important metabolites of *B. fragilis* are short-chain fatty acids (SCFAs), which act as a link between the microflora and immune system by activating GALT cells through free fatty acid receptors (FFARs) [47].

*Bacteroides thetaiotaomicron* and *Clostridium ramosum*, which were studied by Sefik et al. [48], are two individual bacterial species capable of inducing RORγ+ Treg activation. RORγ+ Tregs constitute the main subgroup of Tregs in the colon and differentiate in response to bacterial antigens 15–20 days after birth [47]. Mice with RORγ + Treg deficiency demonstrate microbial dysbiosis, an increase in inflammatory Th17 cells, and are more susceptible to colitis in various models [49,50,51].

#### 2.1.2. Induction of Th1 Cells by Microorganisms and Their Consortia

So far, only a very limited number of commensal species have been described that stimulate a non-inflammatory homeostatic Th1 response. One study identified *Klebsiella*, which is typically present in the oral cavity but can ectopically colonize the gut under conditions of dysbiosis and induce a strong Th1 response [52]. This response was pro-inflammatory and dependent on CD11b−CD103+ expression [53].

#### 2.1.3. Induction of Th17 Cells by Microorganisms and Their Consortia

Segmented filamentous bacteria (SFB) are one of the main inducers of Th17 cells. Mice without SFB showed reduced levels of Th17 when compared with genetically identical mice colonized by a microbiota containing these bacteria [54]. Th17s express a surface TCR specific for SFB antigens. Epithelial adhesion of SFB stimulates the production of reactive oxygen species (ROS) and serum amyloid A (SAA), which play a significant role in the differentiation of Th17 cells [55]. Thus, epithelial adhesion of SFB induces activation of SFB-specific Th17 cells.

Why does SFB mainly induce Th17 cell differentiation? A key feature of SFB is a close association with epithelial cells in the intestine. Atarashi and colleagues, by using mouse-specific strains of SFB, found that only strains derived from the original host were able to cause induction of Th17 cells due to species-specific adhesion [56]. SFB adhesion induced the expression of serum amyloid proteins SAA1/2 and reactive protein dual oxidase 2 (Duox2), resulting in reactive oxygen species (ROS) production in epithelial cells. SAA1 enhances the induction of IL-17A and IL-17F expression by Th17 cells and also affects DCs by promoting Th17 activation and enhancing RORγt + expression. In addition, the action of IL-22, a canonical cytokine of Th17s, is increased and effectively upregulates antimicrobial peptides. Upregulation of antimicrobial peptides causes the appearance or disappearance of bacteria such as *C. rodentium* and some pathogenic strains of *S. typhimurium, E. coli,* and *Yersinia enterocolitica* [54]. Colonization of SFB enhances the expression of MHC class II on intestinal epithelial cells and affects enterocyte glycosylation. These changes in glycosylation often result in expression of glycolipids GM1 (GM1 gangliosidosis), purported to inhibit the attachment of another adherent microbe [57,58]. According to new data, SFB has also been identified in humans [59,60]. Symbiotic bacteria *Bifidobacterium adolescentis* induce a strong population of Th17 cells in the gut without provoking inflammation of the mucous membrane [61].

#### 2.1.4. Induction of T Follicular Helper (T_FH_) Cells by Microorganisms and Their Consortia

In Peyer’s patches (PPs), T_FH_ cells support B cell maturation in germinal centers and their differentiation into plasma IgA-secreting cells [62]. Extracellular ATP produced by microbes regulates T_FH_ cell activation, which in turn controls the level of secretory IgA [63]. *A. muciniphila* has been shown to induce antigen-specific T_FH_ cells to stimulate antigen-specific, T-dependent production of IgA and IgG_1_ [64]. SFB induced PPs T_FH_ cell differentiation by inhibiting the IL-2 signaling pathway in PPs. Despite recent attention on the T_FH_ cell field, little is known regarding the interaction of commensals and T_FH_ cells [65].

#### 2.1.5. Induction of T lymphocytes by Microbial Metabolites

##### Short-Chain Fatty Acids

**Figure 2:**
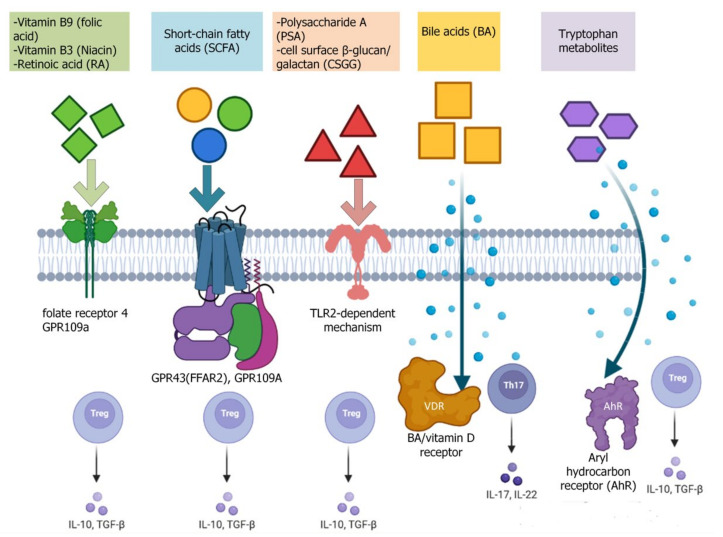
Induction of T-lymphocytes by microbial metabolites. Shown here are the main mechanisms of intestinal CD4+ T cell induction by major microbial metabolites.

Three major SCFAs produced by gut bacteria are acetate, propionate, and butyrate [66]. These are aliphatic carboxylic acids containing 1 carbon in the carboxylic function and 1, 2, and 3 carbons in the aliphatic tail, respectively [66]. Several groups of bacteria can produce acetate, including those belonging to clusters IV and XIVa *Clostridia* species, and are also an important source of butyrate in the intestine [67]. Propionate is formed by the bacterial species *Bacteroides* and *Firmicutes* [67]. Butyrate and acetate have been shown to promote induction and function of intestinal Tregs by several mechanisms—e.g., by enhancing Foxp3 expression in CD4 T cells via increased histone acetylation [68]. The effects of SCFAs are mediated by binding to FFARs, such as G protein-coupled receptors (GPCRs) expressed on CD4+ T cells (GPR43, also known as FFAR2) and DCs (GPR109A) [69]. Unlike SCFAs, long-chain fatty acids enhance the differentiation and proliferation of Th1 and Th17 cells [70] (Figure 2).

##### Adenosine Triphosphate (ATP)

Luminal ATP has been shown to promote differentiation of intestinal Th17 cells and can be obtained from both the host and the microbiota [47]. ATP derived from commensal bacteria can activate a subset of lamina propria cells, CD70^high^CD11c^low^, leading to the differentiation of Th17 cells [71]. Systemic or rectal administration of ATP into germ-free mice results in a marked increase in the number of lamina propria Th17 cells. A CD70^high^CD11c^low^ subset of the lamina propria cells express IL-6, IL-23p19, and TGF-β-activating integrin-αV and -β8, in response to ATP stimulation, preferentially inducing Th17 differentiation. The critical role of ATP is further underscored by the observation that administration of ATP exacerbates a T-cell-mediated colitis model with enhanced Th17 differentiation [72].

##### Microbial Polysaccharides

A good example of a bacterial compound with immunomodulatory activity is polysaccharide A (PSA), produced by *B. fragilis* [73]. PSA was reported to prevent colitis in mice [74]. This protective effect of PSA (via *B. fragilis*) was mediated by production of IL-10 from the intestinal Tregs in a TLR2-dependent mechanism [75], as well as by indirect effects on conventional [76] or plasmacytoid DCs [77]. In addition, *B. fragilis* has been shown to release PSA through outer membrane vesicles (OMVs) transported to DCs [76,78]. These OMV-primed DCs protected animals from experimental colitis through induction of intestinal Tregs.

Mannan/β-1,6-glucan-containing polysaccharides (MGCP) are capable of exerting potent anti-inflammatory effects on the immune system. MGCP facilitates Treg induction from naive T cells through a unique Dectin1–Cox2 signaling axis in DCs. Furthermore, through a TLR2-dependent mechanism, it restrains Th1 differentiation of effector T cells by suppressing IFN-γ expression [79].

##### Vitamins

Retinoic acid (a metabolite of vitamin A1) promotes Treg induction and inhibits the development of Th17 cells [80]. Folate receptor 4, a vitamin B9 receptor, is highly expressed on the surfaces of Treg cells, implying a specific role for this vitamin in regulation of Treg cell function [81]. In the absence of folic acid (vitamin B9), naive T cells can differentiate into Tregs. However, these differentiated Treg cells fail to survive due to a concomitant decrease in expression of anti-apoptotic molecules (e.g., Bcl-2). As a result, mice maintained on a vitamin B9-deficient diet have decreased numbers of intestinal Treg cells. Consequently, the impaired survival of Treg cells in these mice leads to their increased susceptibility to intestinal inflammation [82]. Niacin (vitamin B3) signals through the G-protein-coupled receptor (GPR) 109a. This interaction induces anti-inflammatory properties, including the expression of retinal dehydrogenases in colon macrophages and DCs, which in turn trigger Treg differentiation. GPR109a-deficient mice contain reduced colonic Tregs and heightened susceptibility to colonic inflammation [83]. Thus, vitamin B3 promotes colonic Treg generation and maintains colon homeostasis [84].

##### Bile Acids

Bile acids (BAs) function as signaling molecules with pleiotropic metabolic and immune effects through interaction with host receptors and the microbiota. Primary BAs are synthesized from cholesterol in the liver and conjugated to either taurine or glycine prior to secretion into bile. After a meal, BAs are expelled into the small intestine to facilitate fat absorption. BAs are efficiently maintained within the enterohepatic circulation by ileal and hepatic transporter systems. However, 5% to 10% of BAs that are not reabsorbed can serve as substrates for microbial metabolism and undergo biotransformation to secondary BAs. The major biotransformations include hydrolysis of conjugated BAs to free BAs and glycine or taurine by bile salt hydrolase (BSH); 7α-dehydroxylation of cholic acid (CA) and chenodeoxycholic acid (CDCA), yielding deoxycholic acid (DCA) and lithocholic acid (LCA), respectively; and BA 7β-dehydroxylation of ursodeoxycholic acid (UDCA), yielding LCA [85,86,87].

Mice fed a high-fat diet showed altered conjugated BA composition, leading to increases in the sulfate-reducing bacterium *Bilophila wadsworthia*. These changes were associated with enhanced intestinal Th1 responses and the development of colitis in genetically susceptible IL-10-deficient animals [88]. The BA metabolite isoallolithocholic acid enhances the differentiation of anti-inflammatory Treg cells by facilitating the formation of a permissive chromatin structure in the promoter region of the transcription factor Foxp3 [89].

### 2.2. The Role of Intestinal Microbes in Pulmonary Diseases, Gut–Lung Axis, and Changes in Intestinal Microbiota in COVID-19 Patients

Research by Xu et al. demonstrated patients with COVID-19 had a reduction in genera such as *Bifidobacterium and Lactobacillus* [90]. In another study, Zuo et al. examined the intestinal microbiota of 15 patients with SARS-CoV-2 infection by collecting fecal samples 2–3 times during their hospital stay [91]. They found a decreased number of commensal bacteria (*Eubacterium ventriosum, Faecalibacterium prausnitzii*, *Roseburia,* and *Lachnospiraceae*) and an increased amount of opportunistic pathogens (*Clostridium hathewayi, Actinomyces viscosus,* and *Bacteroides nordii*). Microorganisms such as *Coprobacillus, Clostridium hathewayi*, and *Clostridium ramosum* have also been linked to disease severity [92] (Table 1). The same authors found that COVID-19 patients had significantly reduced microbial diversity, more opportunistic bacteria (i.e., *Streptococcus*, *Rothia*, *Veillonella*, *Actinomyces)*, and a reduction in the number of beneficial symbionts. Yeoh et al. utilized DNA sequencing to analyze the intestinal microbiota of 100 hospitalized patients with COVID-19 (34 received antibiotics, 73 received antivirals) and found that intestinal dysbiosis was connected with disease severity in patients receiving antibiotics [93]. Additionally, in patients with COVID-19 disease, there was a significant reduction in the number of *Actinobacterium*, *Bifidobacterium adolescentis*, *Faecalibacterium prausnitzii*, and *Eubacterium rectale* in comparison with healthy controls [93]. A number of these bacteria also correlated with the severity of COVID-19 [93].

Lv et al. studied the connection between changes in the intestinal microbiota and clinical signs of COVID-19 in 67 hospitalized COVID-19 patients. Here, the authors found a significant reduction in the number of fungi *Chromista, Mucoromycota, Ascomycota*, and *Basidiomycota* compared with uninfected people. *Mucoromycota* positively correlates with microorganisms, such as *Intestinibater, Peptostreptococcaceae*, *Aspergillus, Agathobacter*, and *Fusicatenibacter*.

Also noteworthy are the studies of Tang et al., which examined the intestinal microbiota of 57 COVID-19 patients and stratified results depending on disease severity. In patients with mild COVID-19, a negative correlation of C-reactive protein (CRP) with *C. butyricum* was observed. In the severe group, *C. leptum* and *F. prausnitzii* positively correlated with the concentration of neutrophils, and *E. rectale* positively correlated with the IL-6 concentration. In patients with a critical course of COVID-19, levels of *C. butyricum* negatively correlated with the concentration of CRP, and *Bifidobacterium* negatively correlated with prothrombin time and LDH [94].

**Figure 3:**
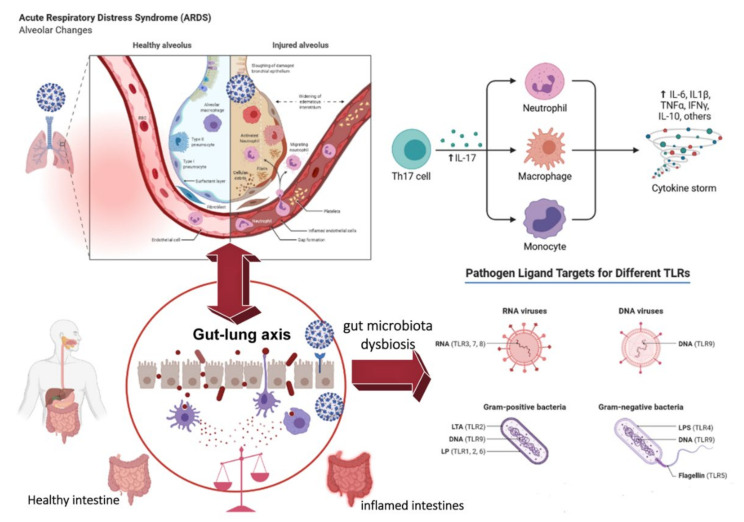
The gut–lung axis in COVID-19.

It has been established that intestinal microbiota affects lung status through cross-interaction via the gut–lung axis [102] (Figure 3). The gut–lung axis is bidirectional, meaning that microbial metabolites and endotoxins can affect the lungs, and simultaneously, lung tissue inflammation can affect the intestinal microbiota [103]. This idea is, to some extent, consistent with studies researching intestinal dysbiosis in COVID-19.

An example of microbiota influence on the gut–lung axis is seen when SFB in the gut stimulates the lung Th17 response, protecting it from *Streptococcus pneumoniae* infection and enhancing lung mucosal immunity [104]. Dysbiosis of intestinal flora has been associated with respiratory diseases such as asthma and cystic fibrosis [105,106]. One study showed endogenous *Bifidobacteria* spp. intestinal flora caused by a fatal influenza infection can enhance resistance to the virus [107]. Conversely, lung inflammation can affect intestinal flora. For example, influenza virus infection in mice can increase the number of *Enterobacteriaceae* and reduce the number of *Lactobacillus* spp. and *Lactococcus* spp. in the gut [103,108,109,110,111]. Another example is that after lipopolysaccharide (LPS) administration to mice, the resultant dysbiosis of the lung microbiota will be accompanied by disturbance of the intestinal flora as bacteria moves from the lungs into the bloodstream [103,108,109,110,111]. These studies have shown that microbes play an essential role in the cross-talk between the gut and the lungs and that microbial dysbiosis in the lungs may affect gut homeostasis and vice versa [111].

The gut–lung axis is the link between the intestinal microbiota and the lungs. Dysbiosis can affect inflammation of lung tissue due to inhibition of anti-inflammatory Tregs and induction of pro-inflammatory Th17s by bacterial metabolites. The result is a cytokine storm, which is a severe complication of COVID-19.

### 2.3. The Role of Intestinal Microbiota in Type 2 Diabetes Mellitus

The intestinal microbiota is connected to the pathogenesis of numerous chronic illnesses, including T2D. Changes in the intestinal microbiota composition in T2D are shown in Table 2.

The most common microorganisms with potential antidiabetic effects are *Bacteroides* and *Bifidobacterium* spp. *Bifidobacterium* is considered by most studies an antidiabetic microorganism [112,113,116,117]. The antidiabetic properties of *Bifidobacterium infantis*, *Bifidobacterium pseudocatenulatum*, *Bifidobacterium bifidum*, *Bifidobacterium animalis*, *Bifidobacterium longum*, and *Bifidobacterium breve* have also been demonstrated by improved glucose tolerance in various animal models [118,119,120,121]. Similar properties were observed for the genus Bacteroides—namely, *Bacteroides acidifaciens* [122] and *Bacteroides uniformis* [123]. *Akkermansia muciniphila* is another protective organism against the development of diabetes. A negative correlation was shown between increasing amounts of this bacterium and the course of T2D [124,125].

The study by Eckburg et al. demonstrated that the intestinal microbiota of people with T2D was characterized by an increase in *Bacteroides* spp., *E. coli*, and *Desulfovibrio* spp. [126]. Conversely, healthy controls demonstrated an increase in butyrate-producing bacteria—namely, *Clostridium* spp., *Eubacterium rectale*, and *F. prausnitzii* [126].

In comparison with their healthy counterpart, Kim-Anne Lê et al. found a reduction in the number of *Bifidobacteria* alongside an increased amount of *Enterococcus* in the intestines of T2D patients [127].

Decreased butyrate levels lead to reduced pancreatic secretion, resulting in lowered levels of glucagon, insulin, and insulin sensitivity [128]. Furthermore, elevated acetic acid levels can activate the parasympathetic nervous system, stimulating the islets of Langerhans beta cells to secrete more insulin, increasing food intake and the development of T2D diabetes [129].

### 2.4. Changes in the Gut Microbiota in Patients with Type 2 Diabetes Receiving Metformin

With the recent development of next-generation (high-throughput, deep) sequencing (NGS) technology, more research has begun focusing on metformin effects on the intestinal microbiota. Table 3 summarizes ten studies on metformin effects on the intestinal microbiota in obese or T2D patients, two of which were performed in obese patients [130,131] and eight in patients with T2D [132,133,134,135,136,137,138,139].

Summarizing and comparing the results in rodent models and human patients, metformin significantly increased the number of bacteria from phylum *Verrucomicrobia*—namely, *Akkermansia muciniphila* [140]. This bacteria is the only species of the phylum Verrucomicrobia present in humans [140]. *Akkermansia muciniphila* is one of the well-known utilizers of mucin, which is decreased in patients with diabetes, obesity, and cardiovascular disease not treated with metformin. It plays a vital role in maintaining intestinal mucosa integrity, reducing the migration of pro-inflammatory LPS, and improving blood glucose levels [141,142,143,144]. Another clinical study of 27 healthy young adults (the participants were given metformin up to 1g twice daily) found that metformin reduced *Clostridium* spp. and *Intestinibacter* spp., while the number of *Escherichia/Shigella* spp. and *Bilophila wadsworthia* increased [145]. The main mechanisms of metformin’s effect on the intestinal microbiota are summarized below.

#### 2.4.1. Maintaining the Integrity of the Intestinal Barrier

The interaction between the intestinal barrier integrity and the gut microbiota is very important in the development and progression of metabolic diseases. Shin et al. found an increased level of LPS induced by a high-fat diet in serum is inhibited by metformin and suggested this was due to the changes in the intestinal microbiota composition [146]. Similarly, metformin protected intestinal barrier function and reduced blood LPS levels in a mouse model of insulin resistance and obesity [147]. In addition, exogenous administration of LPS blocked the enhancement of blood glucose control and insulin signaling caused by metformin. These results indicate that metformin might improve glucose metabolism by maintaining intestinal barrier function [148].

#### 2.4.2. Enhancement of SCFAs Synthesis

SCFAs produced by the gut microbiota can exert beneficial effects on peripheral tissues, such as adipose tissue, skeletal muscle, and liver, by controlling substrate metabolism and function to improve insulin sensitivity. The ability of the gut microbiota to produce butyrate and propionate is enhanced in patients treated with metformin. Metformin treatment may result in an increased number of SCFA-producing bacteria, such as *Akkermansia, Butyricicoccus, Ruminococcus, Phascolarctobacterium, Coprococcus, Allobaculum*, *Bacteroides*, *Blautia*, *Lactobacillus*, and *Butyricimonas* [149,150]. Metformin has also been shown to enhance active and total glucagon-like peptide 1, which is consistent with the hypothesis that it increases SCFA production through modification of the gut microbiota composition [151,152].

#### 2.4.3. Regulation of Bile Acid Metabolism

Improving glucose metabolism with metformin treatment was shown by its ability to regulate the total serum BAs of diabetic rats. Wu et al. showed a significant increase in plasma BAs during 4-month metformin treatment, while BAs in fecal samples remained unchanged. The level of BSH produced by the intestinal microbiota increased in the second month of metformin treatment [117]. Sun et al. analyzed the gut microbiota in patients newly diagnosed with T2D who were treated for the first time with a 3-day metformin regimen. They found metformin increased the level of glycoursodeoxycholic acid by regulating the gut microbiota (such as inhibition of *Bacteroides fragilis* growth) [134]. In turn, regulation of the gut microbiota inhibited the farnesoid X receptor signaling pathway to reduce blood glucose and maintain blood glucose homeostasis [134]. Finally, it was found that metformin reduced the reabsorption of BA in the distal ileum, leading to increased bile salt concentration within the colon [153]. Herein lies a potential explanation for the effects of metformin on the colonic microbiota [153].

### 2.5. Discussion and Conclusions

The intestinal microbiota is a complex consortium of microorganisms affecting most systems of the human body. Undoubtedly, one of the most important tasks of the intestinal microbiota is the modulation of immune cells. Representatives of the intestinal microbiota can activate different subpopulations of T lymphocytes in the intestinal plate, both directly and indirectly through metabolites, which may ultimately lead to enhancement and suppression of certain T lymphocyte subpopulations.

COVID-19 has become a major challenge for society and science. The emerging data suggest that immune system dysregulation could be a link connecting COVID-19 infection severity, diabetes, and the microbiota [154]. Researchers found not only a link between the influence of the intestinal microbiota on the immune system, but also a direct effect on the lungs, which was called the gut–lung axis. The mesenteric lymphatic system is an important communication route between the intestine and the lungs. It allows intact bacteria, their fragments, or metabolites to translocate through the intestinal barrier, reach the circulatory system, and modulate the lung’s immune response [155]. For example, SCFAs, which are mainly synthesized through the fermentation of bacterial dietary fibers, act as signaling molecules in the lungs in cells presenting resident antigens, thereby reducing inflammatory and allergic responses [156,157].

Diabetes and older age are both risk factors for severe disease and increased mortality in patients with COVID-19 [158]. Indeed, the case fatality rate for patients with no comorbidities is approximately 0.9%, but it rises to 7.3% for those with diabetes [159]. The chronic inflammatory condition in T2D is considered the underlying mechanism for the “cytokine storm” complication and worsens the outcomes of COVID-19 [160]. It is assumed that hyperinflammation may result in multiorgan failure, a general complication in critical COVID-19 cases [161].

In recent years, it has been reported that T cells play key roles in the progression of T2D. This included T2D association with overactivated T cells and subsequent activation of inflammatory pathways [162]. Additionally important has been an immune response in T2D patients defined by the balance between Treg and Th1 or Th17 cells. Tregs suppress the activities of Th1, Th2, and Th17 cells to improve insulin resistance. Moreover, it has been described that the percentage of Tregs was decreased in peripheral blood of T2D patients, especially in newly diagnosed patients [163,164,165]. This culminated in inflammation and insulin resistance [163,164,165]. Similarly, it was reported that both the Treg/Th17 ratio and Treg/Th1 ratio decreased in patients suffering from T2D [166,167]. These intestinal immunomodulatory cells are a peculiar bridge between the intestinal microbiota and diabetes [168].

When compared with healthy controls, T2D patients commonly have a decreased amount of SCFA-producing bacteria (*Eubacterium rectale*, *Faecalibacterium prausnitzii*, *Roseburia intestinalis*, *Roseburia inulinivorans*, *Akkermansia*, and *Bifidobacterium*) and tryptophan metabolite-producing bacteria (*Lactobacillus*, *Bacteroides*, *Bifidobacterium*, *Peptostreptococcus*, *Ruminococcus*, *Ruminiclostridium*, and *Clostridium*) [124,169,170,171,172,173,174]. There is also a concurrent increase in the amount of opportunistic pathogens (*Bacteroides caccae* and *Clostridium hathewayi*), branch chain amino acid synthesizing bacteria (*Bacteroides vulgatus* and *Prevotella copri*), and sulfate-metabolizing bacteria (*Desulfovibrio*, *Lactobacillus gasseri*, and *Lactobacillus reuteum*) compared with healthy controls [124,169,170,171,172,173,174]. Since SCFAs regulate Th17s/Tregs, the reduction in SCFA-producing bacteria in T2D may be related to COVID-19-induced hyperinflammation through the gut–lung axis [175].

In the review, we document research describing changes to gut microbiota in two important worldwide diseases: COVID-19 and T2D. Nonetheless, the question of how the microbiota changes during comorbidity remains unresolved. In our opinion, dysbiosis in T2D may influence the course of COVID-19 through the gut–lung axis. Decreased number of SCFA-producing bacteria during T2D may regulate the level of Tregs and, consequently, could be a contributing factor in the development of the cytokine storm.

Given the proposed interplay between COVID-19 and T2D, the use of metformin is promising. In addition to its hypoglycemic action, it can affect the intestinal microbiota, leading to increased production of microbial metabolites such as SCFAs that induce Treg cells, which, in turn, modulates excessive immune responses. This property of metformin is not well-publicized and has important therapeutic potential. Metformin also has possible additional antiviral activity against SARS-CoV-2 infection via activation of the protein kinase by AMP (AMPK), leading to the phosphorylation of ACE2 and resulting in conformational and functional changes in the surface protein. This may lead to a subsequent increase in ACE2 expression via AMPK that may lead to a decrease in SARS-CoV-2 binding [176].

## Figures and Tables

**Table 1:** COVID-19 and gut microbiota changes.

Research	Study Design	Number of Participants	Research Methods	Microbiota Changes
Zuo et al. [91]	Single-center, prospective	15 COVID-19 patients compared against 6 subjects with community-acquired pneumonia and 15 healthy individuals	Metagenomic sequencing	Significant changes in the microbiota of the GI tract (dysbiosis) in patients with COVID-19. Positive correlation between the severity of COVID-19 and dysbiosis.
Gu et al. [92]	Single-center, cross-sectional	30 COVID-19 patients compared against 24 H1N1 patients and 30 matched healthy controls	16S rRNA sequencing	Significantly reduced diversity of bacteria (dysbiosis), significantly lower relative number of beneficial symbionts, and higher relative number of opportunistic pathogens in COVID-19.
Yeoh et al. [93]	Prospective cohort study from two centers	100 COVID-19 patients compared against matched healthy controls	Sequencing of fecal DNA. Assessment of levels of inflammatory markers.	Significant changes in the microbiota of the GI tract (dysbiosis) in COVID-19 patients. Dysbiosis continued even after 30 days post-illness. Significant correlation of dysbiosis with COVID-19 severity and numerous pro-inflammatory markers in serum.
Prasad et al. [95]	Prospective cohort study from one center	30 hospitalized patients with COVID-19 and 16 healthy subjects.	16S rRNA sequencing and markers of intestinal permeability	Abnormal signs of microorganisms were observed in plasma samples from approximately 65% of COVID-19 patients. Compared with the uninfected control group, plasma levels of intestinal permeability markers (such as FABP2, PGN, and LPS) were significantly higher in COVID-19 patients.
Newsome et al. [96]	Prospective cohort study from one center	50 hospitalized COVID-19 patients, 9 recovered patients, and 34 uninfected subjects.	16S rRNA sequencing	The microbial composition of feces differed significantly in COVID-19 patients. Patients with COVID-19 had an increased relative amount of *Campylobacter* and *Klebsiella* spp.
Lv et al. [97]	Prospective cohort study from one center	56 hospitalized COVID-19 patients and 47 healthy subjects.	Metabolomics, gas chromatography	There were differences in the metabolomes of COVID-19 patients compared with uninfected members of the control group.
Tanget et al. [94]	Cohort study	Total: 57 (20 mild hospitalized COVID-19 patients, 19 severe hospitalized COVID-19 patients, 18 critical hospitalized COVID-19 patients)	qPCR	Intestinal dysbiosis progressed depending on the severity of the disease. Significant reduction in the number of probiotic bacteria *Bifidobacterium* and *Lactobacillus* compared with uninfected people. Significant decrease in the number of butyrate-producing bacteria (anti-inflammatory bacteria) *C. leptum, F. prausnitzii*, *E. rectale,* and *C. butyricum* compared with uninfected people.
Chen et al. [98]	Cohort study	30 hospitalized COVID-19 patients	16S rRNA sequencing	At the beginning of the disease, dysbiosis was observed, and it continued throughout the disease course. A correlation was found between the severity of the disease course and the diversity of the intestinal microbiota.
Mazzarelliet et al. [99]	Cohort study	Total: 23 (6 COVID-19 patients in the ICU (i-COVID-19); 9 COVID-19 patients in the infectious disease wards (w-COVID-19), 3 non-COVID-19 hospitalized patients in the ICU, 5 non-COVID-19 patients in general ward)	qPCR	Decreased microbial diversity in ICU-treated COVID-19 patients compared with those treated in the infectious department. Significant increase in opportunistic pathogens *Enterobacteriaceae, Actinobacteria, Proteobacteria*, *Peptostreptococcaceae, Staphylococcaceae*, *Aerococcaceae, Vibrionaceae*, and *Dermabacteraceae* compared with patients without COVID-19. Significant reduction in fusobacteria and spirochetes compared with patients without COVID-19.
Lv et al. [100]	Prospective cohort study from one center	Total: 150 (67 hospitalized COVID-19 patients, 35 hospitalized H1N1 patients, 48 healthy individuals)	qPCR with primers ITS1f and ITS2r	*Aspergillus niger* was common in COVID-19 patients and positively correlated with symptoms of diarrhea. Significant reduction in the number of *Mucoromycota, Basidiomycota, Ascomycota*, and *Chromista,* compared with uninfected people. *Mucoromycota* positively correlates with opportunistic pathogens *Intestinibater, Agathobacter, Peptostreptococcaceae*, *Aspergillus,* and *Fusicatenibacter*. *Penicillium citrinum* was negatively correlated with the concentration of CRP.
Yu et al. [101]	Cohort study	Total: 3 hospitalized COVID-19 patients	Sequencing	Intestinal dysbiosis may be an important factor in severe COVID-19 infection. Significant increase in *Kluyneromyces, Aspergillus, Firmicutes, Actinobacteria,* and *Corynebacterium* in COVID-19 patients compared with uninfected subjects.

**Table 2:** Type 2 diabetes and gut microbiota changes.

Research	Study Design	Number of Participants	Research Methods	Changes in the Intestinal Microbiota	
				Increase	Decrease
Candela et al. [112]	Open-label trial	40 patients with T2D and 13 healthy controls	16S rRNA sequencing	*Enterobacteriaceae*, *Collinsella*, *Streptococcus*, *Lactobacillus*	*Bacteroides*, *Lachnospira*, *Prevotella*, *Roseburia*, *Faecalibacterium*
Sedighi et al. [113]	Case–control study	18 patients with T2D and 18 healthy controls	16S rRNA sequencing	*Lactobacillus*	*Bifidobacterium*
Wu et al. [114]	Double-blind study	16 patients with T2D and 12 healthy controls	16S rRNA sequencing	No data	*B. vulgatus* and *Bifidobacterium*
Larsen et al. [115]	Open-label trial	18 patients with T2D and 18 healthy controls	16S rRNA sequencing	*Bacteroidetes, C. coccoides, Firmicutes*	*Clostridia, Firmicutes*

**Table 3:** Gut microbiota in patients with T2D receiving metformin.

Research	Research Methods	Number of Participants		Dosage of Metformin	Changes in the Intestinal Microbiota	
		Metformin-untreated T2D	Metformin-treated		Increase	Decrease
Forslund et al. [132]	Metagenomics	106	93	No data	*Escherichia*	No data
Cuesta-Zuluaga et al. [133]	16sRNA	14	14	No data	*Prevotella*, *Megasphaera*, *Butyrivibrio*	*Oscillospira*, *Barnesiellaceae*
Wu et al. [117]	Metagenomics	22	22	1700 mg/d	*Pectobacterium*, *Pantoea*, *Serratia*, *Raphidiopsis*, *Dickeya*, *Helicobacter*, *Bacillus, Rheinheimera, Citrobacter, Yersinia, Shewanella*, *Enterobacter, Erwinia*, *Cronobacter*, *Dermacoccus*, *Pseudomonas*, *Salmonella*, *Klebsiella*, *Escherichia*	*Pseudogulbenkiania, Subdoligranulum, Acetivibrio, Bartonella, Dethiosulfovibrio*, *Hippea*, *Pseudoflavonifractor*, *Deferribacter, Intestinibacter*
Hung et al. [135]	qPCR	23	23	No data	*Enterobacteriaceae*	No data
Sun et al. [134]	Metagenomics	22	22	1000 mg/d	No data	*Bacteroides fragilis*
Barengolts et al. [136]	16sRNA	11	21	No data	*Bifidobacterium*, *Catenibacterium*, *Parabacteroides*	No data
Ejtahed et al. [131]	16sRNA	20	20	1000 mg/d	*Escherichia/Shigella*	No data
Zhang et al. [137]	16sRNA	26	51	No data	*Actinobacteria*, *Fusobacteria*, *Betaproteobacteria*	*Gammaproteobacteria*, *Erysipelotrichi*
Hiel et al. [130]	16sRNA	53	42	No data	*Akkermansia*, *Clostridium* cluster XIVa, *Clostridium* cluster XIVb, *Klebsiella, Escherichia/Shigella*	*Roseburia, Clostridium cluster* XI, *Clostridium cluster* XVIII
Chavez-Carbajal et al. [138]	16sRNA	14	14	No data	*Bacteroidales*, *Acidobacteriales*, *Pelomonas* spp.	No data
